# Diskitis as Manifestation of Gait Disturbance

**Published:** 2018

**Authors:** Afagh ASSANZADEHRAD, Vahid AMINZADEH

**Affiliations:** 1 Pediatrics Growth Disorders Research Center, School of Medicine, Guilan University of Medical Sciences, Rasht, Iran.

**Keywords:** Diskitis, Gait disturbance, Limping, Iran

## Abstract

Gait disturbance is a common presentation of neurologic disease in children. Limping is a kind of gait dysfunction that occurs due to neurologic & skeletal diseases. Diskitis is an inflammatory process noted as one of the significant causes of limping especially in children aged less than 3 yr. Here we report case of diskitis and limping as the significant manifestation of Gait disturbance in A 22 months old boy from 17 Shahrivar Hospital in 2016, Rasht, northern Iran. Regarding normal neurologic exam, nervous system involvement was less possible. About 60% of gait cycle related to stance phase.

## Introduction

Children typically begin walking between 12-16 months. Neurologic maturation is necessary for development of gait and there is no difference between 7 years old child and adult regarding gait characteristics (1). Within 55 week after achieving independent gait, children commonly acquired adult patterns of walking (2). According to the complexity of walking process, the cooperation of many motor systems including basal ganglia, sensory cortex, neck proprioceptors, cerebellum, spinal motor & sensory tract, grey matter, peripheral nerve, neuromuscular junction & muscles is mandatory (3,4).

Although gait disturbances can be noted as the usual manifestation of childhood neurologic diseases, clinician must be aware that not all gait abnormalities relate to neurologic dysfunction (5). Limping either painful or painless can be noticed as a kind of gait disturbance. Proximal muscle weakness or hip instability cause painless gait. Patients with painless gait have the equal stance phase in each involved & uninvolved sides. However, for the balance, children must lean or shift the center of gravity over the involved extremity (6).

Bilateral disorders cause waddling gait. Based on patients’ age, various etiologies of abnormal gait disorders can be mentioned: 

1) Toddler 

Painful limp: Septic arthritis, osteomyelitis, toxic synovitis, trauma, occult trauma, malignancy, and diskitis 

Painless limp: Development of dislocation of hip, neuromuscular disease, cerebral palsy, lower extremity length inequality 

2) Children aged 4-10 year

Painful limp: Septic arthritis, osteomyelitis, toxic synovitis, myositis, trauma, rheumatologic disorders, malignancy, and diskitis 

Painless limp: Development of dislocation of hip, Legg- Calve- Perthes disease, neuromuscular disease, lower extremity length inequality, cerebral palsy and Duchenne muscular dystrophy. 

In addition, non-skeletal etiologies such as testicular torsion, inguinal hernia and appendicitis may cause Limping in some cases (1). Early identification of the underlying problem causing a limp can be identified by history & clinical examination. 

Diskitis is the disk space infection, which occurs often in children aged less than 5 yr old secondary to subacute infection of adjacent vertebral body *Staphylococcus aureus* is the leading organism causes diskitis (7). 

As this case was an uncommon and treatable cause of gait disturbance in children and regarding the importance of reporting this issue for educational purpose, we report here diskitis as manifestation of gait disturbance.

## Case presentation

A 22 months old boy referred to 17 Shahrivar Hospital, Rasht, north of Iran in 2016 with the complaint of gait disturbance (painful gait) with limping. Informed consent was obtained from parents. Growth and development were normal. The patient was afebrile with good mental status and with mild irritability, normal active and passive range of motion of pelvis.

**Fig 1:**
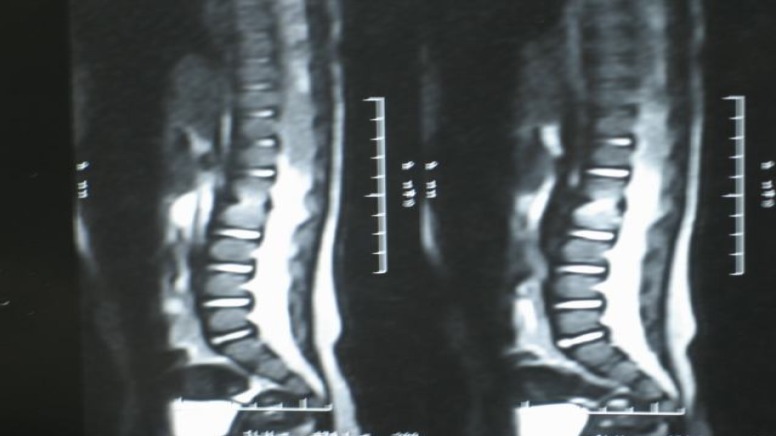
Lumbosacral MRI

Skin over spinal area was normal and there was no spinal deformity. He refused walking. Although he had limping with shortness of stance phase, painful walking was possible. He could not sit and stand because of discomfort. However, in a supine position, no discomfort was mentioned. In addition, deep tendon reflex and the muscle tone in all extremities was normal. 

Lab data were as follows: WBC: 5700 with PMN 50%, ESR in first time: 38 and second time: 85, CRP+3, CPK:38. *Brucella* screen test, ANA, and RF were negative. Brain CT, EMG, NCV (performed in another center) and pelvic sonography showed normal results. In lumbar MRI, hypersignal intensity in L2-L3 space was reported (Figure 1). Therefore, clinician suspected diskitis and immediately administered intravenous cloxacillin for 2 wk and oral cephalexin for other 2 wk. 

The patient had good course and after 7 d, he walked independently. ESR became 25 and CRP: -VE. 

## Discussion

In our case, gait disturbance was the patient complaint that could be mentioned due to many causes. Walking is a complex skill a normal participation of many motor systems is necessary. Regarding normal neurologic exam, nervous system involvement was less possible. Therefore, brain CT, EMG, NCV (performed in another center) were normal. Gait cycle divided into stance & twisting phase and 60% of them related to stance phase. With observation of child, gait limping was noted. In limping, painful or painless gait should be differentiated because of diverse differential diagnosis. 

The patient had painful gait but was afebrile and nontoxic. There was no limitation in the range of motion but there was difficulty in bending posture so toxic synovitis, septic arthritis, osteomyelitis were ruled out. Diskitis should be considered in all children who refuse walking and with normal neurologic finding especially in child less than 3 year. Blood culture and screening tests for rheumatologic diseases were negative. Osteomyelitis of vertebral body should be considered most often in patients aged more than 5 yr old with fever and actually ill patients (1) and in rheumatoid arthritis, there was several joint involvements. Screening for brucellosis was negative. WBC count was normal but increase of ESR and +VE CRP. 

According to clinical suspicious to diskitis, lumbar MRI was done (early diagnosis is possible by lumbar MRI). It revealed diskitis of L2-L3 space and treatment began immediately and early treatment indicated good clinical & para clinical course.
